# Supplementary material for: Chromatin spatial organization of wild type and mutant peanuts reveals high-resolution genomic architecture and interaction alterations
Source: Genome Biol. 2021 Nov 16;22:315. doi: 10.1186/s13059-021-02520-x (PMC8594070; doi:10.1186/s13059-021-02520-x)
Supplement: Supplementary file 2 — Additional file 2. Supplementary figures (Fig. S1 to Fig. S7). [file 13059_2021_2520_MOESM2_ESM.docx]

**Additional file 2 (Fig S1 to Fig S7)**


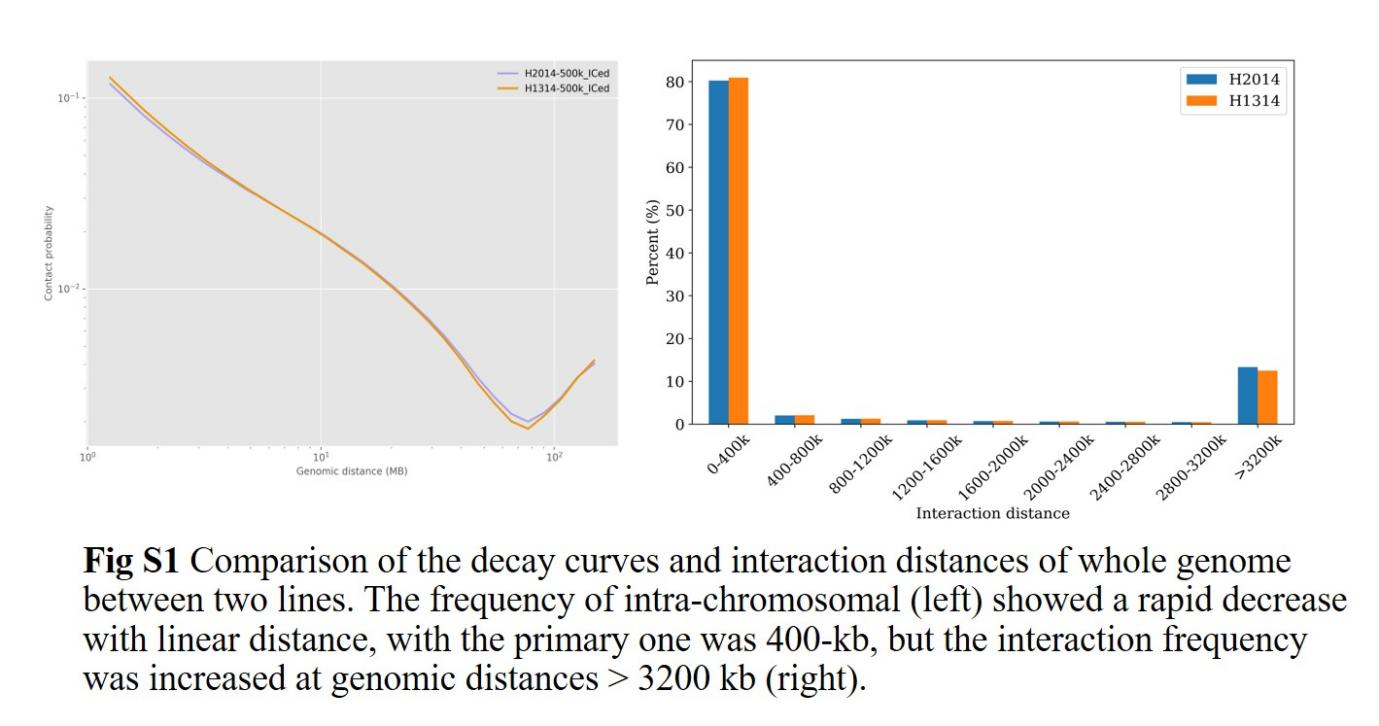


**Figure S1:** **Comparison of the decay curves and interaction distances of whole genome between two lines.** The frequency of intra-chromosomal (left) showed a rapid decrease with linear distance, with the primary one was 400 kb, but the interaction frequency was increased at genomic distances >3200 kb (right).


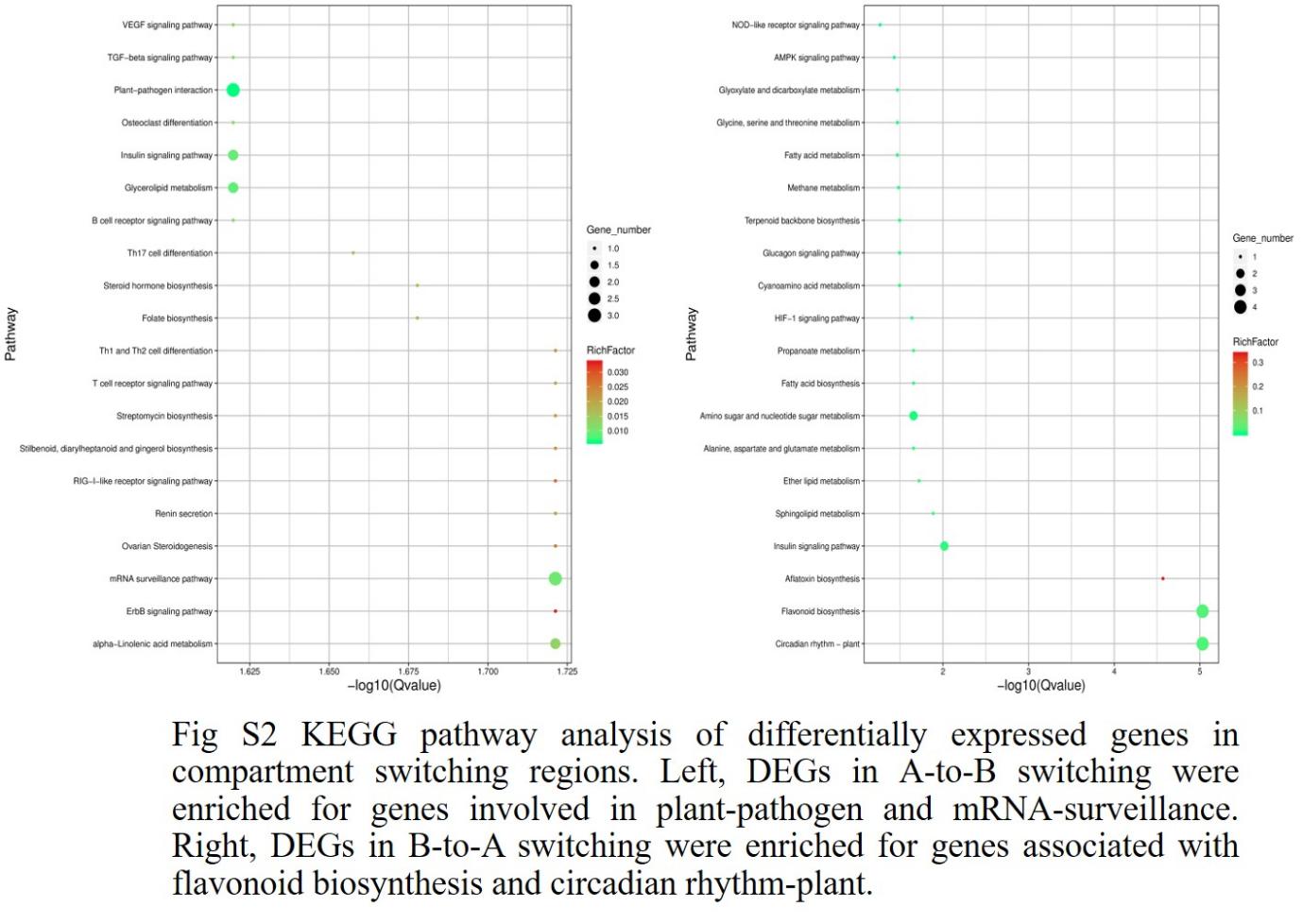


**Figure S2:** **KEGG pathway analysis of differentially expressed genes in compartment switching regions.** DEGs in A-to-B switching (left) were enriched for genes involved in plant-pathogen and mRNA-surveillance. DEGs in B-to-A switching (right) were enriched for genes associated with flavonoid biosynthesis and circadian rhythm in plants.


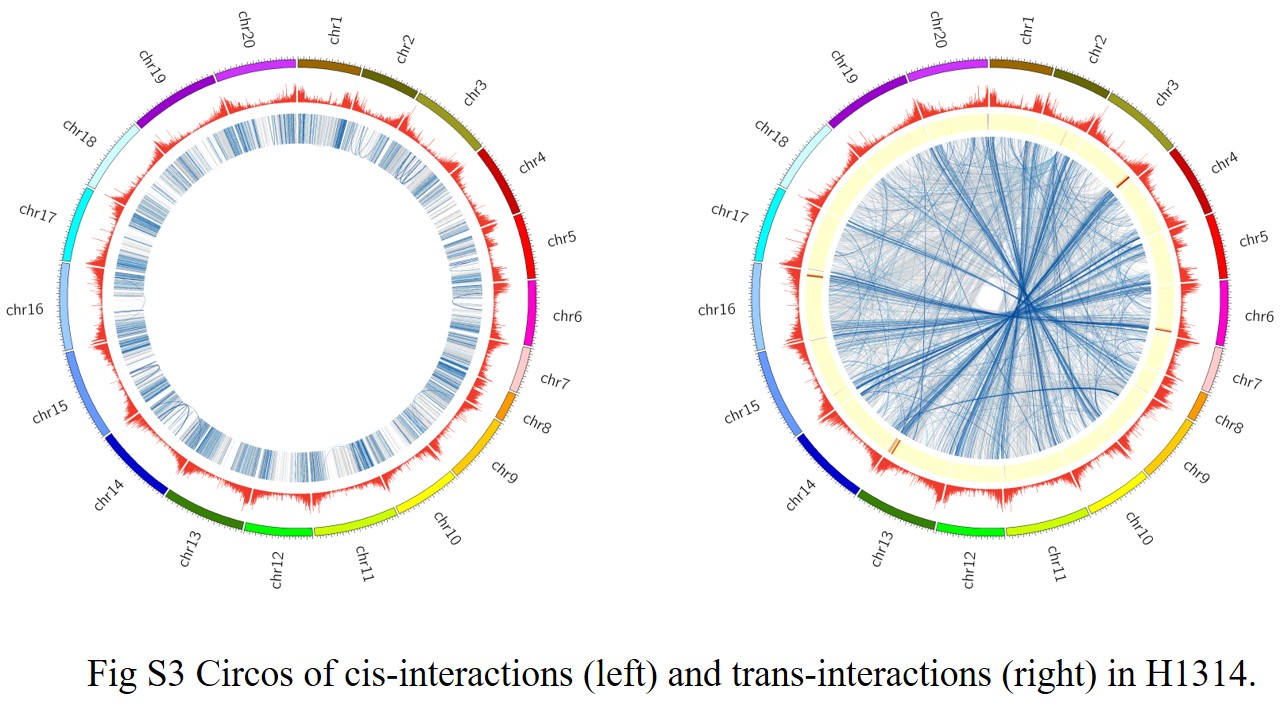


**Figure S3:** **Circos of cis-interactions (left) and trans-interactions (right) in H1314.**


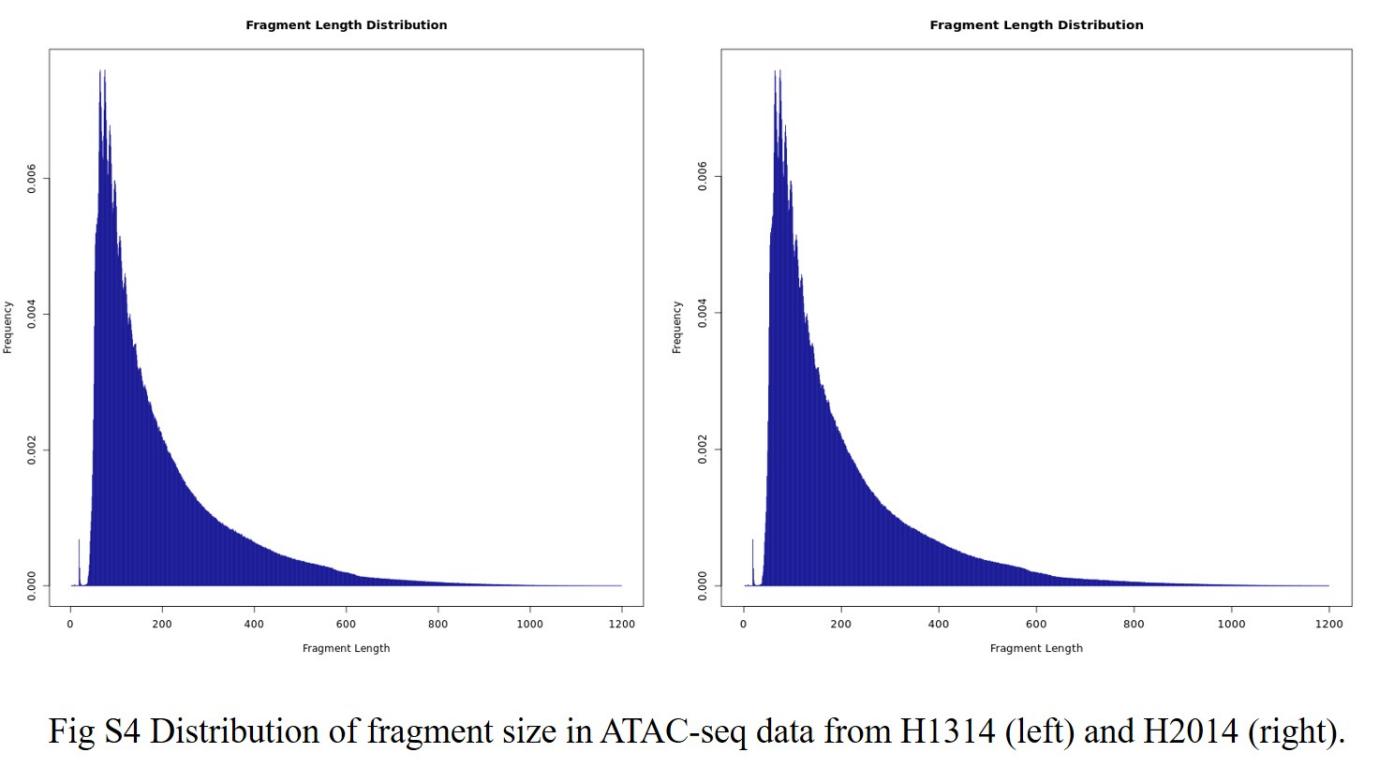


**Figure S4:** **Distribution of fragment size in ATAC-seq data from H1314 (left) and H2014 (right).**


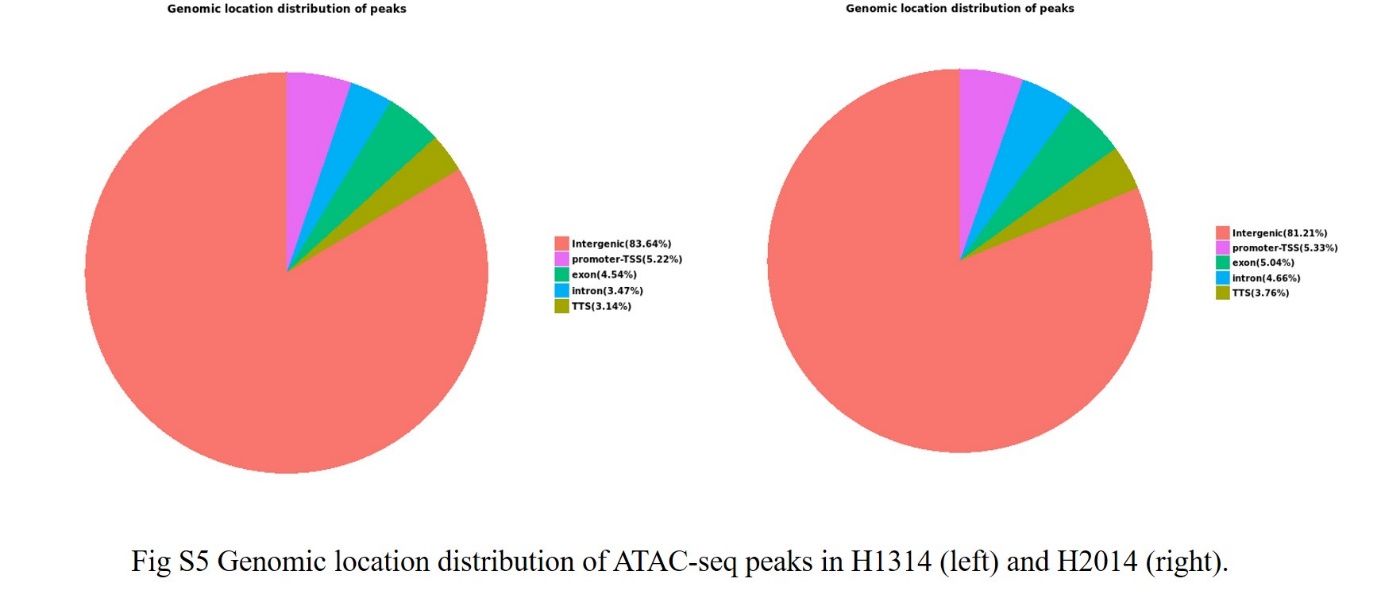


**Figure S5:** **Genomic location distribution of ATAC-seq peaks in H1314 (left) and H2014 (right).**


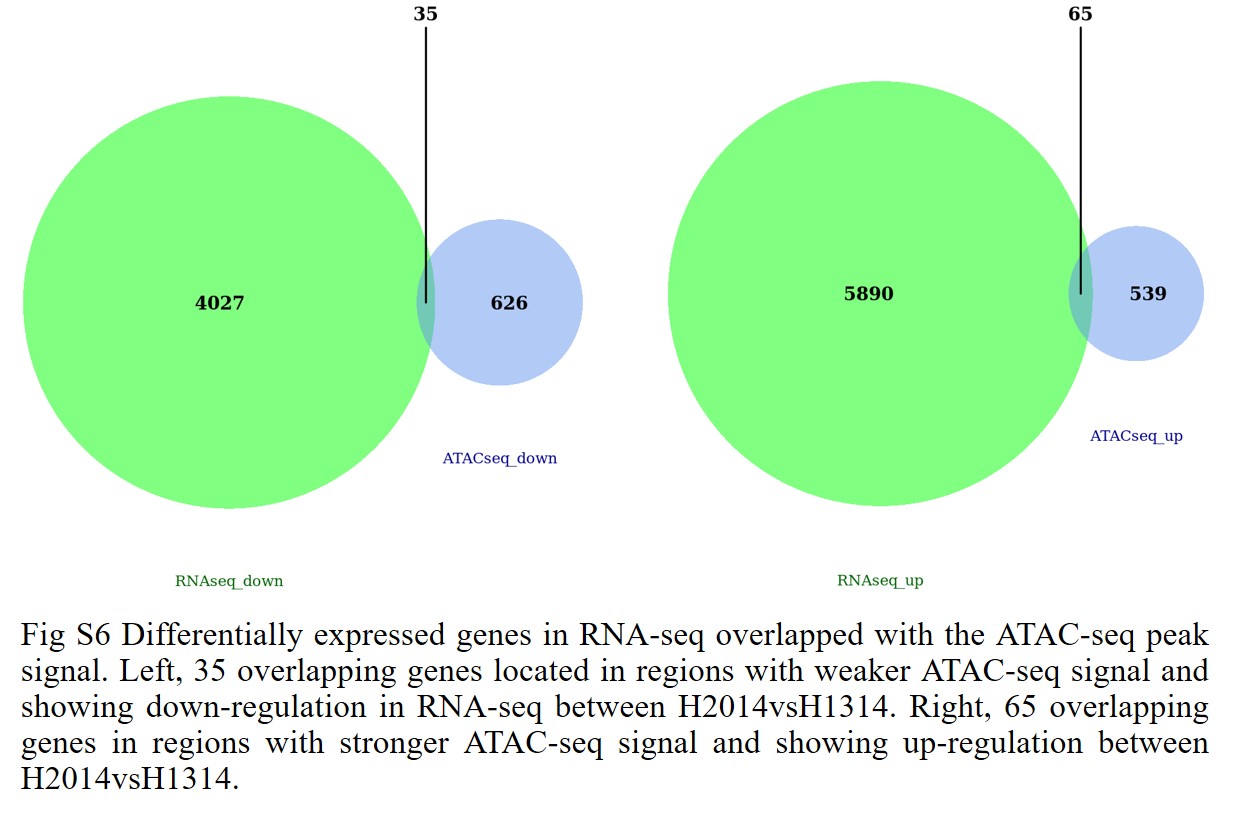


**Figure S6:** **Differentially expressed genes in RNA-seq overlapped with the ATAC-seq peak signal.** Total 35 overlapping genes located in the regions with weaker ATAC-seq signal and showing down-regulation in RNA-seq between H2014 vs H1314 (left). Total 65 overlapping genes in regions with stronger ATAC-seq signal and showing up-regulation between H2014 vs h1314 (right).


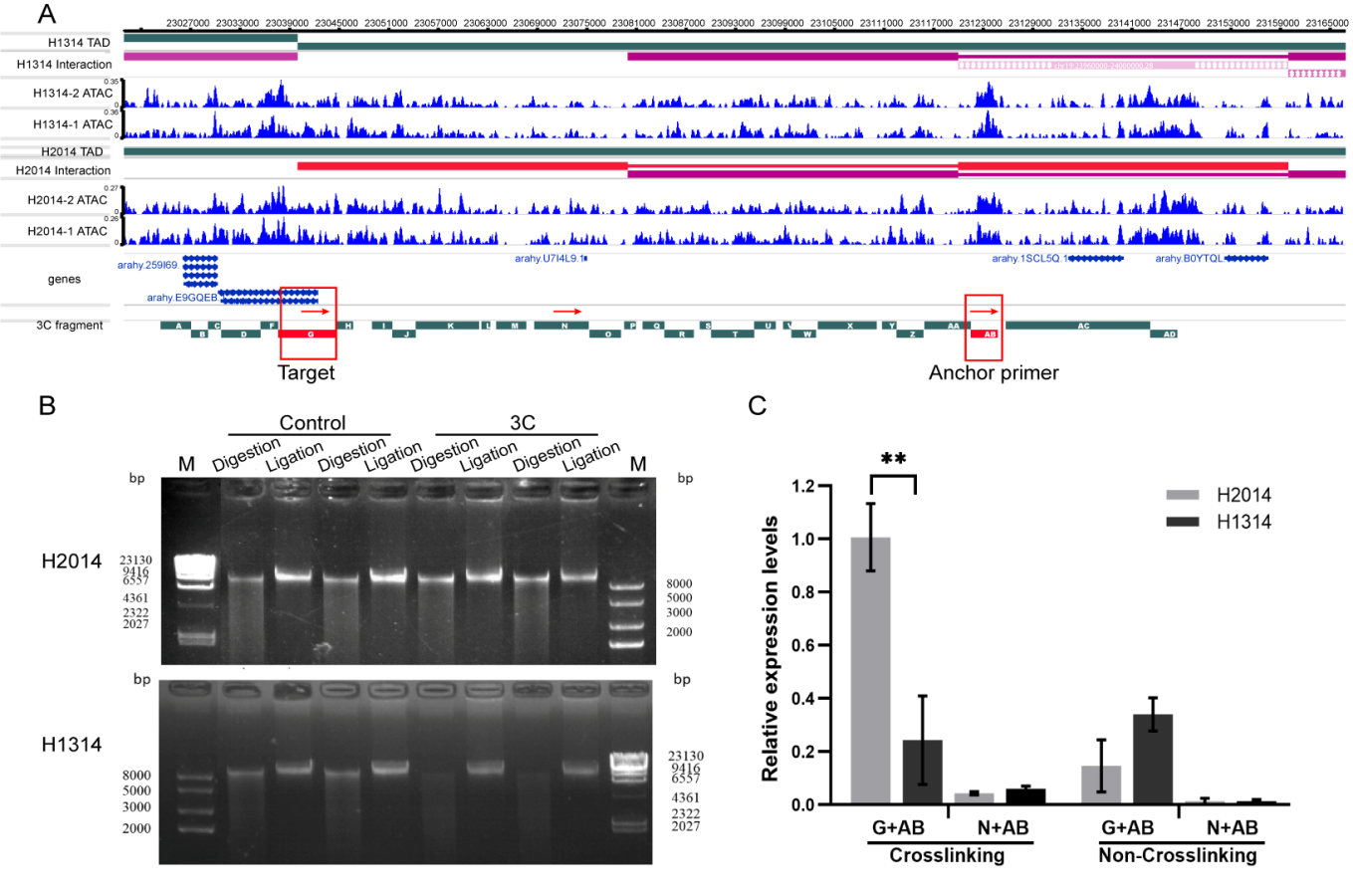


**Figure S7:** **Validation of the chromatin loop on the chr19.** A, Diagram of the genomic region on chr19 (22,950,000 – 23,360,000) showing the TADs, and chromatin interactions, and ATAC peaks in the dwarf mutant (H1314) and the wild type (H2014). The digested fragments with length longer than 1 kb (green boxes tracked with letter) were used to design 3C-qPCR primers. The anchor regions corresponding to AB and G primers were used to examine the chromatin loop (marked with red boxes). B, Gel imaging of the 3C experiments in H2014 (top) and H1314 (bottom). For panels, “Control” and “3C” represent non-crosslinked and crosslinked samples, respectively. C, 3C-qPCR examining the chromatin loop in H2014 and H1314. Crosslinked and non-crosslinked samples were analyzed with the anchor primer set (G+AB), and the PCR with primer set (N+AB) was served as a low interaction frequency control. Error bars represent SDs from three biological repeats. ** *P* < 0.01.
